# Genome-wide copy number alteration and *VEGFA* amplification of circulating cell-free DNA as a biomarker in advanced hepatocellular carcinoma patients treated with Sorafenib

**DOI:** 10.1186/s12885-019-5483-x

**Published:** 2019-04-01

**Authors:** Chung Ryul Oh, Sun-Young Kong, Hyeon-Su Im, Hwa Jung Kim, Min Kyeong Kim, Kyong-Ah Yoon, Eun-Hae Cho, Ja-Hyun Jang, Junnam Lee, Jihoon Kang, Sook Ryun Park, Baek-Yeol Ryoo

**Affiliations:** 1 Department of Internal Medicine, Asan Medical Center, University of Ulsan College of Medicine, Seoul, Republic of Korea; 2 Department of Laboratory Medicine, Hospital, National Cancer Center, Goyang, Republic of Korea; 3 Department of Clinical Epidemiology and Biostatistics, Asan Medical Center, University of Ulsan College of Medicine, Seoul, Republic of Korea; 4 Cancer Biomedical Science, Graduate School of Cancer Science and Policy, Goyang, Republic of Korea; 5 College of Veterinary Medicine, Konkuk University, Seoul, Republic of Korea; 6 Genome Research Center, Green Cross Genome, Yongin, Republic of Korea; 7 Division of Hematology-Oncology, Department of Medicine, Samsung Medical Center, Sungkyunkwan University School of Medicine, Seoul, Republic of Korea; 8 Department of Oncology, Asan Medical Center, University of Ulsan College of Medicine, 88 Olympic-ro, 43-gil, Songpa-gu, Seoul, 05505 Republic of Korea

**Keywords:** Hepatocellular carcinoma, Circulating cell-free DNA, Sorafenib, Biomarker, Genome-wide copy number alteration, Vascular endothelial growth factor-a

## Abstract

**Background:**

Although sorafenib is the global standard first-line systemic treatment for unresectable hepatocellular carcinoma (HCC), it does not have reliable predictive or prognostic biomarkers. Circulating cell-free DNA (cfDNA) has shown promise as a biomarker for various cancers. We investigated the use of cfDNA to predict clinical outcomes in HCC patients treated with sorafenib.

**Methods:**

This prospective biomarker study analyzed plasma cfDNA from 151 HCC patients who received first-line sorafenib and 14 healthy controls. The concentration and VEGFA-to-EIF2C1 ratios (the VEGFA ratio) of cfDNA were measured. Low depth whole-genome sequencing of cfDNA was used to identify genome-wide copy number alteration (CNA), and the I-score was developed to express genomic instability. The I-score was defined as the sum of absolute Z-scores of sequenced reads on each chromosome. The primary aim of this study was to develop cfDNA biomarkers predicting treatment outcomes of sorafenib, and the primary study outcome was the association between biomarkers with treatment efficacy including disease control rate (DCR), time to progression (TTP) and overall survival (OS) in these patients.

**Results:**

The cfDNA concentrations were significantly higher in HCC patients than in healthy controls (0.71 vs. 0.34 ng/μL; *P* < 0.0001). Patients who did not achieve disease control with sorafenib had significantly higher cfDNA levels (0.82 vs. 0.63 ng/μL; *P* = 0.006) and I-scores (3405 vs. 1024; *P* = 0.0017) than those achieving disease control. The cfDNA-high group had significantly worse TTP (2.2 vs. 4.1 months; HR = 1.71; *P* = 0.002) and OS (4.1 vs. 14.8 months; HR = 3.50; *P* < 0.0001) than the cfDNA-low group. The I-score-high group had poorer TTP (2.2 vs. 4.1 months; HR = 2.09; *P* < 0.0001) and OS (4.6 vs. 14.8 months; HR = 3.35; *P* < 0.0001). In the multivariable analyses, the cfDNA remained an independent prognostic factor for OS (*P* < 0.0001), and the I-score for both TTP (*P* = 0.011) and OS (*P* = 0.010). The VEGFA ratio was not significantly associated with treatment outcomes.

**Conclusion:**

Pretreatment cfDNA concentration and genome-wide CNA in cfDNA are potential biomarkers predicting outcomes in advanced HCC patients receiving first-line sorafenib.

**Electronic supplementary material:**

The online version of this article (10.1186/s12885-019-5483-x) contains supplementary material, which is available to authorized users.

## Background

Primary liver cancer is a deadly malignancy, with 782,500 new cases and 745,500 deaths reported worldwide in 2012 [1]. Liver cancer ranks 2nd and 6th highest as the cause of cancer-related death in men and women, respectively, and remains an important public health issue in the world [1]. Hepatocellular carcinoma (HCC) is the most common type of primary liver cancer and accounts for approximately 75–90% of all liver cancers. [1, 2] Advanced unresectable HCC is among the most difficult-to-treat cancers because of its resistance to systemic chemotherapy and underlying liver dysfunction. Systemic chemotherapy was not recommended until 2007, when the molecular targeted agent sorafenib, an inhibitor of vascular endothelial growth factor (VEGF) receptor, platelet-derived growth factor receptor, Raf family kinases, and other tyrosine kinases, demonstrated survival benefits in advanced HCC patients [3, 4]. Although sorafenib is the global standard first-line systemic treatment for advanced unresectable HCC, it does not have reliable predictive or prognostic biomarkers [3, 4]. Several studies suggested the potential biomarkers include soluble c-Kit and hepatocyte growth factor in plasma, and *VEGFA* amplification in tumor tissues as predictive markers [5, 6], or alpha-fetoprotein (AFP), alkaline phosphatase, angiopoietin 2, VEGF, and neutrophil-to-lymphocyte ratio in the blood as prognostic markers [5, 7]; however, these biomarkers have not been validated or translated into clinical practice. Recent data reported that *VEGFA* could promote tumor development and growth in a preclinical model of HCC and suggested *VEGFA* genomic amplification in HCC tumor tissues as a predictive biomarker for sorafenib based on results showing survival of patients with HCC who did not receive sorafenib was independent of *VEGFA* status in tumor tissue, whereas markedly improved survival was seen in the *VEGFA*-amplification group compared with the non- amplification group in sorafenib-treated patients [6, 8].

Circulating tumor DNA (ctDNA) has the potential to reveal tumor genetic and epigenetic information while overcoming obstacles related to tumor heterogeneity and clonal evolution; thus cfDNA holds great promise as a liquid biopsy. Given that HCC is frequently diagnosed using radiologic imaging without pathologic confirmation, and biopsy for this cancer is associated with a relatively high risk of bleeding risk for biopsy, ctDNA in the peripheral blood would be especially useful in HCC. Previous studies have reported that the presence of ctDNA reflected tumor progression after surgery in HCC, and high cfDNA concentration was associated with larger tumors, higher tumor grade, and shorter overall survival after surgery, and may serve as a predictive biomarker for distant metastasis after curative surgery in HCC [9, 10]. However, there are no data about the prognostic role of cfDNA concentrations in the setting of advanced HCC treated with systemic treatment.

To develop novel cfDNA-based biomarkers as predictors of outcome in HCC patients treated with sorafenib, we evaluated cfDNA concentration itself and genetic alterations in cfDNA focusing on *1)* a specific gene, *VEGFA* amplification based on previous data suggesting *VEGFA* amplification in tumor tissue as a potential biomarker for sorafenib [6, 8], and *2)* genome-wide copy number alterations (CNAs).

## Methods

### Study aim

The primary aim of this study was to develop cfDNA biomarkers predicting disease control rate (DCR), time to progression (TTP), and overall survival (OS) in patients who had advanced or metastatic HCC not amenable to local therapies and were treated with first-line sorafenib.

### Study design and population

This prospective biomarker study was performed in the subpopulation who received first-line sorafenib among the entire study population in an open-label, exploratory, observational, biomarker study in patients who had advanced or metastatic HCC not amenable to local therapies and were treated with systemic therapy. Longitudinal blood samples ± tissue samples including baseline samples before treatment were prospectively collected in eligible patients.

This study was conducted under approval from the Institutional Review Board at Asan Medical Center, Korea (IRB No. 2014–1208). Patients were included in this study if they met the following criteria: 1) age ≥ 18 years; 2) histologically or radiologically confirmed advanced or metastatic HCC not amenable to local therapies; 3) first-line treatment with sorafenib; 4) measurable or evaluable lesion(s) according to the Response Evaluation Criteria In Solid Tumors (RECIST) version 1.1 [11]; and 5) available peripheral blood samples obtained before the start of sorafenib for cfDNA analysis. Exclusion criteria were as follows: 1) fibrolamellar HCC, sarcomatoid HCC, or mixed cholangiocarcinoma and HCC; 2) prior systemic treatment for HCC; 3) concurrent other malignancy; and 4) no available imaging study for evaluation of response to sorafenib. All patients provided written informed consent before study enrollment. Clinical data of patients were prospectively collected.

Plasma samples from 14 healthy volunteers were used as negative controls and were collected after obtaining signed informed consent from each patient.

### Treatment and assessment

Patients received sorafenib 400 mg twice a day, and dose reduction was allowed at the discretion of the physician. Treatment was continued until progressive disease (PD), patient withdrawal, or unacceptable toxicity.

Tumor response was evaluated using computed tomography according to RECIST version 1.1 every 6–8 weeks. DCR was defined as the percentage of patients with best tumor response of complete response (CR), partial response, or stable disease (or non-CR/non-PD in the case of non-measurable disease). OS was defined as the time from initiation of sorafenib to death from any cause, and TTP was defined as the time until radiologic disease progression, respectively.

### Blood sample collection and cfDNA extraction

Peripheral blood samples from patients before starting sorafenib or healthy donors were collected in EDTA tubes and centrifuged within 4 h at room temperature at 1600×g for 10 min first, and then 3000×g for 10 min to isolate the plasma, which was then stored at − 80 °C until cfDNA extraction. Plasma cfDNA was extracted from 1.5 mL of plasma from each patient with the QIAamp Circulating Nucleic Acid kit (Qiagen, Hilden, Germany) following the manufacturer’s instructions. The final DNA eluent (50 μL) was quantified by Qubit 2.0 Fluorometer with the qubit dsDNA HS (High Sensitivity) assay kit (Life Technology, Carlsbad, CA, USA).

### Detection of *VEGFA* amplification

*EIF2C1* was used as a reference to assess the copy number of the *VEGFA* gene because it is known to be expressed at ubiquitously at low to medium levels. Plasma VEGFA-to-EIF2C1 ratios (the VEGFA ratio) were determined using droplet digital polymerase chain reaction (ddPCR) on a QX200 Droplet Digital PCR System (Bio-Rad Laboratories). Fluorescent probes (FAM and HEX) were prepared from PrimePCR™ ddPCR™Copy Number Assay for ddPCR (dHsaCP2500483 for VEGFA and dHsaCP2500349 for EIF2C1) (Bio-Rad Laboratories, Pleasanton, CA, USA).

Each sample was partitioned into 20,000 droplets, and target and control (background) DNA were randomly, but uniformly, distributed among the droplets. Reactions were performed in 20 μL reaction volumes that consisted of extracted cfDNA (8 μL), 2× ddPCR supermix for the probe (10 μL), and 20× VEGFA and EIF2C1 probe (FAM/HEX) (1 μL). The reaction samples and generator oil are placed into a QX200 droplet generator, which uses specially developed reagents and microfluidics to partition each sample into 20,000 nanoliter-sized droplets. The generated droplets are transferred to a 96-well plate for PCR in a thermal cycler. Emulsified PCR reactions in a 96-well plate were run on an Eppendorf Mastercycler nexus gradient Thermal Cycler (Master Cycler, Eppendorf, Germany) at 95 °C for 10 min, followed by 40 cycles of 94 °C for 30 s, 55 °C for 60 s, and a 10 min incubation at 98 °C. The plates were read on a Bio-Rad QX200 droplet reader (Bio-Rad, Hercules, CA, USA) using the QuantaSoft v1.4.0 software (Bio-Rad) to assess the number of droplets positive for VEGFA and EIF2C1.

### Library preparation for whole-genome sequencing

The DNA libraries were prepared using the TruSeq nano kit (Illumina Inc., San Diego, CA, USA). Briefly, approximately 5 ng of cfDNA was subjected to end repair, adenylation, and adaptor ligation. High sensitivity D1000 Screen Tape (Agilent Technologies, Santa Clara, CA, USA) was used to examine the size distribution of the final libraries. The pooled libraries of 24 samples per run were analyzed with the NextSeq 500 (Illumina Inc.) in a 75-base single-read mode.

### Data analysis for calculation of genome instability

All generated reads were aligned to the human reference genome (hg19) using the BWA-mem algorithm (0.7.5.a) with default parameters [12]. Then, Picard (v.1.9.6) tools (https://broadinstitute.github.io/picard/) were used to remove PCR duplicates. The reads, which were below the mapping quality of 60, were not used for further analysis. The autosomal genome was divided into 1 Mb bins. Of 2897 bins, 163 were not used because these bins were located in low mapping regions such as the centromere and telomere. GC bias correction using the LOESS algorithm was performed for 2734 bins [13]. The GC-corrected read counts for each bin were determined, and the percentage of sequencing reads mapped to each bin was calculated and compared with the mean value of the 14 healthy control subjects for the respective bin. A Z-score statistic was calculated using each bin’s mean and standard deviation (SD). Zj values represent the Z-score of the specific bin, which can be expressed using the following formula:

$$ {\boldsymbol{Zscore}}_{\boldsymbol{bin}}=\frac{\boldsymbol{Normalized}\ \boldsymbol{percentage}\ \boldsymbol{of}\ \boldsymbol{r}\mathbf{e}\boldsymbol{ad}\ \boldsymbol{count}\ \boldsymbol{in}\ \boldsymbol{the}\ {\boldsymbol{bin}}_{\boldsymbol{sample}}-\boldsymbol{Mean}\ \boldsymbol{normalized}\ \boldsymbol{percentage}\ \boldsymbol{of}\ \boldsymbol{read}\ \boldsymbol{count}\ \boldsymbol{in}\ \boldsymbol{the}\ {\boldsymbol{bin}}_{\boldsymbol{control}}}{\boldsymbol{SD}\ \boldsymbol{of}\ \boldsymbol{normalized}\ \boldsymbol{percentage}\ \boldsymbol{of}\ {\boldsymbol{read}\ \boldsymbol{count}}_{\boldsymbol{control}}} $$

To express whole genomic instability (chromosomal instability), we developed the I-score, which is the sum of the absolute Z-scores of all usable bins with Z-score > 2 or < − 2. The I-score is defined as follows:

$$ \mathbf{I}={\sum}_{\boldsymbol{j}\ \boldsymbol{from}\ \boldsymbol{all}\ \boldsymbol{usable}\ \boldsymbol{autosome}\ \boldsymbol{bins}}\mid {\boldsymbol{Z}}_{\boldsymbol{j}}\mid >2 $$

As a surrogate marker of whole genome instability, higher I-score means higher chromosomal instability. I-score is expected to be zero in the normal persons without any cancer.

### Statistical analysis

The primary study outcome was the association between biomarkers and treatment efficacy including DCR, TTP, and OS. The Mann-Whitney test and the chi-square test were used for continuous variable data and categorical data, respectively. Kaplan-Meier method and log-rank test were used to estimate and compare TTP and OS of patients according to the level of cfDNA biomarkers (high vs. low cfDNA concentration; high vs. low I-score; high vs. low *VEGFA* amplification). We dichotomized the level of cfDNA biomarkers into high- and low-groups based on the median value of each biomarker. In the case of I-score, patients were also divided into four quartiles based on I-score values. Patients who did not have events (disease progression for TTP and death for OS) were censored at their last tumor assessment for TTP and at the last follow-up for OS. Univariable analyses were performed to analyze the associations of cfDNA biomarkers and clinicopathological parameters with TTP and OS, and multivariable Cox regression was performed to evaluate the effect of cfDNA biomarkers on TTP and OS, after adjusting for clinicopathological parameters that were statistically significant in the univariable analysis. Hazard ratio (HR) and 95% confidence intervals (CIs) for variables included in the multivariable model were reported. All *P* values reported were two-sided, and *P* values < 0.05 were considered statistically significant.

## Results

### Patient characteristics

**Fig. 1:**
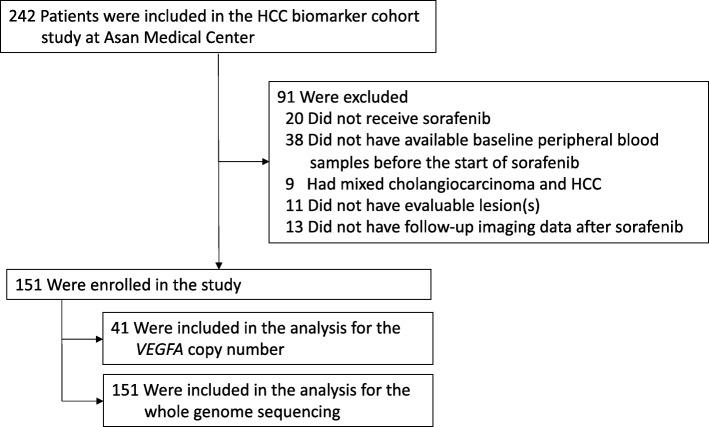
Patients flow chart for the study

Among 242 patients who were enrolled in the advanced or metastatic HCC biomarker study between March 2014 and November 2016, 91 patients were excluded due to not receiving sorafenib as first-line therapy (*n* = 20), absence of available baseline blood samples before sorafenib (*n* = 38), absence of follow-up imaging data after sorafenib (*n* = 13), absence of evaluable lesion(s) (*n* = 11), and mixed cholangiocarcinoma and HCC (*n* = 9), leaving 151 patients eligible for this analysis (Fig. 1). Baseline characteristics are described in Table 1. Most patients had hepatitis B virus infection-associated HCC with Barcelona Clinic Liver Cancer stage C, Child-Pugh Class A liver function, and Eastern Cooperative Oncology Group performance status 0–1.

**Table 1:** Patient characteristics

Characteristics	N = 151
Age, years	57 (52–63)
Sex	
Male	137 (90.7%)
Female	14 (9.3%)
ECOG performance status	
0	52 (34.4%)
1	97 (64.2%)
2	2 (1.3%)
Etiology	
Hepatitis B	134 (88.7%)
Hepatitis C	4 (2.6%)
Alcohol	7 (4.6%)
Others	6 (4.0%)
Child-Pugh class	
A	140 (92.7%)
B	11 (7.3%)
BCLC stage	
B	5 (3.3%)
C	146 (96.7%)
Macrovascular invasion	
Yes	63 (41.7%)
No	88 (58.3%)
No. of extrahepatic spread organ sites	
0	15 (9.9%)
1	79 (52.3%)
2	41 (27.2%)
≥ 3	16 (10.6%)
Sites of extrahepatic spread	
Lymph node	64 (42.4%)
Lung	77 (51.0%)
Bone	32 (21.2%)
Peritoneum	23 (15.2%)
Adrenal gland	13 (8.6%)
AFP (ng/mL)	
< 20	41 (27.1%)
20–200	32 (21.2%)
> 200	77 (51.0%)
Not available	1 (0.7%)
Platelet count (× 10^3^/mm^3^)	122.0 (85.0–165.0)
Prothrombin time (INR)	1.08 (1.02–1.16)
Albumin (g/dL)	3.7 (3.4–4.0)
Total bilirubin (mg/dL)	0.7 (0.5–1.0)
AST (IU/L)	39 (28–58)
ALT (IU/L)	26 (18–39)
Previous therapy	
No	10 (6.6%)
Yes	141 (93.4%)
Surgical resection	69 (45.7%)
RFA	37 (24.5%)
TACE	118 (78.1%)
Radiotherapy	79 (52.3%)
Liver transplantation	12 (7.9%)

Data are the median (interquartile range) or number (%) unless otherwise indicated

*ECOG* Eastern Cooperative Oncology Group, *BCLC* Barcelona Clinic Liver Cancer, *AFP* alpha fetoprotein, *INR* international normalized ratio, *AST* aspartate aminotransferase, *ALT* alanine aminotransferase, *RFA* radiofrequency ablation, *TACE* transcatheter arterial chemoembolization

### Total concentration, *VEGFA* amplification, and genome-wide CNAs in plasma cfDNA

**Fig. 2:**
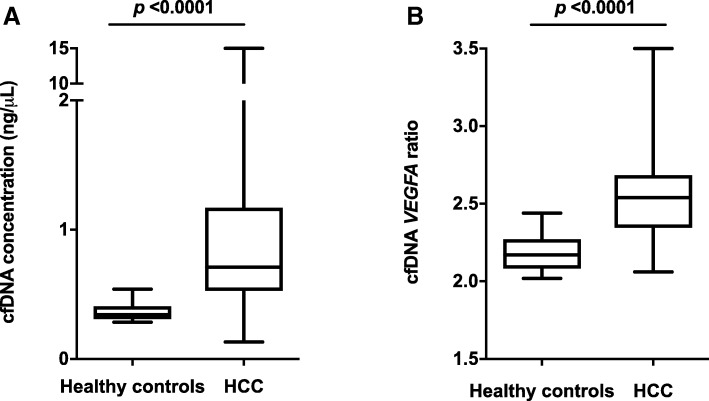
(**a**) Total cfDNA concentration and (**b**) VEGFA ratio in healthy controls and HCC patients. A two-tailed Mann-Whitney U test was performed to compare the median values. The horizontal line in the middle of each box indicates the median, and the top and bottom borders of the box mark the 75th and 25th percentiles, respectively. The whiskers above and below the box mark the ranges. Abbreviations: *cfDNA*, cell-free DNA; *VEGFA*, vascular endothelial growth factor-A; *HCC*, hepatocellular carcinoma

The median cfDNA concentration was 0.71 ng/μL (range, 0.13–15.00) in HCC patients (*n* = 151) and 0.34 ng/μL (range, 0.28–0.54) in healthy controls (*n* = 14) (*P* < 0.0001) (Fig. 2 a). The cfDNA concentrations were significantly higher in HCC patients than in healthy controls (*P* < 0.0001). Elevated cfDNA concentration was observed in 122 patients (80.8%; 95% CI, 74.5–87.1%) compared with the 90th percentile of healthy controls.

In a calibration experiment using cancer cell lines with *VEGFA* amplification (OE19), *VEGFA* amplification was robustly detected with a copy number of 9 to 10 (median, 9.7; range, 9.3–10.4). Although the *VEGFA* copy number was measured only in part of the HCC cohort (*n* = 41) and in healthy controls, it was significantly higher in HCC patients than in healthy controls (median, 2.50 [range, 2.06–3.50] vs. 2.17 [range, 2.02–2.44], respectively; *P* < 0.0001) (Fig. 2b).

**Fig. 3:**
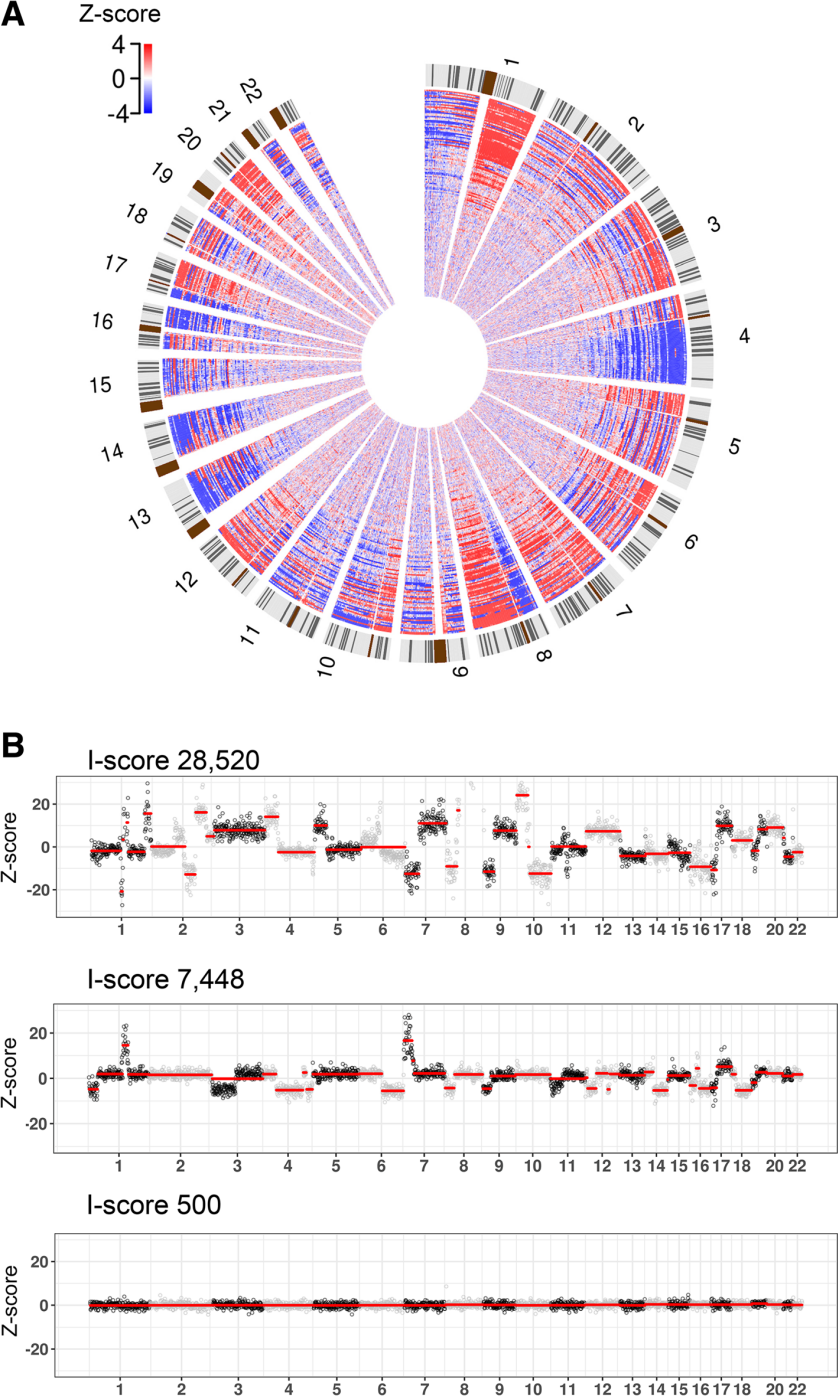
CNA profiles for hepatocellular carcinoma cfDNA. (**a**) Circos plot of the distribution of CNA in the chromosomes of 151 patients. The chromosome map is located on the external periphery with the centromere in blue. The relative chromosomal deviations of individual cfDNA samples from the means of reference samples, expressed as Z-scores (red represents gain; blue represents loss) are illustrated as inner wheels. (**b**–**c**) Representative I-score profiles of three patients. Each point represents the normalized read count ratio of a 1 Mb-sized bin. Separate chromosomes from 1 to 22 are shown, and a Z-score of zero corresponds to a copy number of 2. Abbreviations: *CNA*, copy number alteration; *cfDNA*, cell-free DNA

Whole-genome sequencing was successful in all 151 HCC patients, with a median I-score of 1637 (range, 256–28,520). A Circos plot from 151 HCC patients in which the number of regions significantly deviated from euploidy is illustrated in Fig. 3a. Most frequent chromosomal arm alterations included copy number losses in 1p, 4q, and 8p, and gains of 1q and 8q. GISTIC analysis [14] identified significantly recurrent focal amplifications at 1q21.3(harboring MCL1), 7q31.2 (harboring MET), 8q24.21 (harboring MYC), 11q13.3(harboring CCND1 and FGF19), 13q34, 19p13.11, and deletions at 4q35.2, 8p21.2, 13q14.2(harboring RB1), 14q24.2, 17p13.1(harboring TP53) and 19p13.3 regions. Oncogenes and tumor suppressor genes are included in such recurrent regions which were previously known as significantly altered regions in HCC [15].

Deletion of chromosome 1p, 4q and 8p and gain of chromosome 1q and 8q were frequently observed. Although the I-score significantly correlated with the cfDNA concentration (*P* < 0.0001), the degree of correlation was not high (*R*^*2*^ = 0.24) (Additional file 1: Figure. S1). CNA profiles were expressed as linear genomic plots for three representative patients in Fig. 3b and d.

### Association between *VEGFA* copy numbers and treatment outcomes

Although there was no patient who had CR, two patients (1.3%) achieved PR, and 94 patients (62.3%) had SD or non-CR/non-PD as best response to sorafenib, resulting in 63.6% of the DCR. DCR did not significantly differ between the VEGFA-high group (above the cohort median; 52.6% [95% CI, 30.1–75.1%]) and the VEGFA-low group (68.2% [95% CI, 48.7–87.7%]) (*P* = 0.309). Similarly, TTP did not differ between the VEGFA-high group (3.8 months; 95% CI, 1.5–6.0) and the VEGFA-low group (3.5 months; 95% CI, 2.5–4.5) (*P* = 0.781) (Additional file 1: Figure. S2a). Although the median OS was shorter in the VEGFA-high group (7.5 months; 95% CI, 3.2–11.8) than in the VEGFA-low group (12.8 months, 95% CI, 7.7–18.0), the difference was not statistically significant (*P* = 0.180) (Additional file 1: Figure. S2b). We defined *VEGFA* amplification as a higher value than “mean + 3 x standard deviation” of *VEGFA* copy number in healthy controls, and cut-off value was 2.60. When we analyzed treatment outcomes according to the *VEGFA* amplification, TTP and OS did not significantly differ between the amplification group (*n* = 16) and the non-amplification group (*n* = 25); the median TTP values were 3.8 months (95% CI, 1.1–6.5) and 3.5 months (95% CI, 2.1–4.9) (*P* = 0.725), respectively, and the median OS was 8.4 months (95% CI, 6.4–10.3) and 12.6 months (95% CI, 7.1–18.0), respectively (*P* = 0.626). The DCR was also not different between the two groups; 56.3% in the amplification group vs. 64.0% in the non-amplification group (*P* = 0.620).

### Association between concentration or CNAs in cfDNA and treatment outcome

**Fig. 4:**
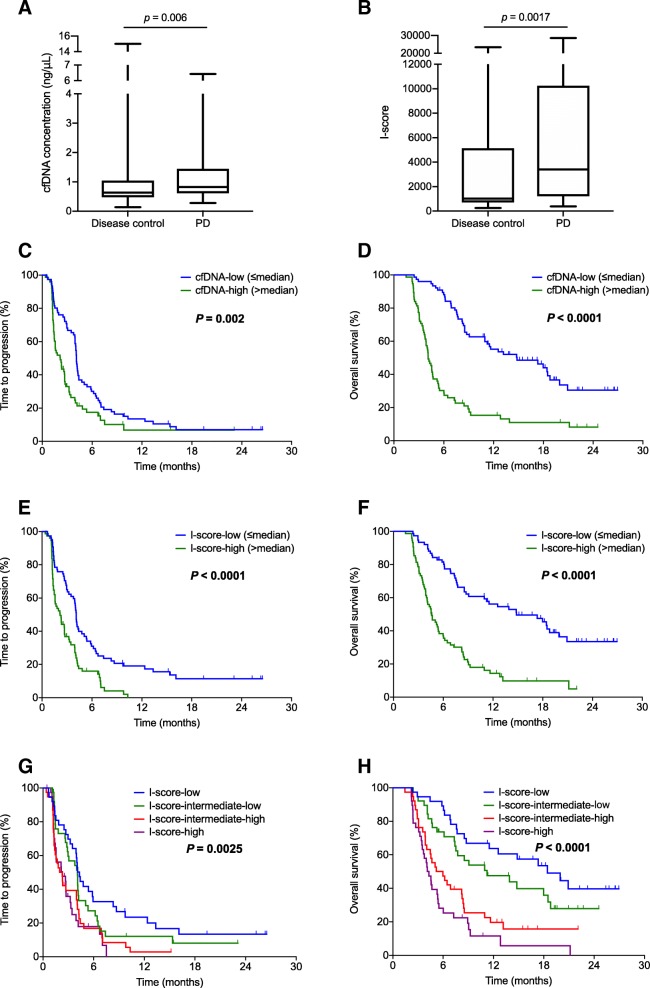
Treatment outcomes according to the cfDNA level and I-score. Comparison of (**a**) the cfDNA level and (**b**) the I-score between patients who achieved disease control and patients who did not. (**c**–**h**) Kaplan–Meier for (**c**) TTP and (**d**) OS according to high vs. low cfDNA level; and (**e**) TTP and (**f**) OS to high vs. low I-score; and (**g**) TTP and (**h**) OS according to I-score quartile. Abbreviations: *cfDNA*, cell-free DNA; *TTP*, time to progression; *OS*, overall survival; *PD*, progressive disease

Patients who did not achieve disease control had significantly higher cfDNA levels than those who did; the median levels were 0.82 ng/μL (range, 0.28–6.42) and 0.82 ng/μL (range, 0.28–6.42) vs. 0.63 ng/μL (range, 0.13–15.0), respectively (*P* = 0.006) (Fig. 4a). The cfDNA-high group (above the median; *n* = 75) had a significantly lower DCR than the cfDNA-low group (*n* = 76) (52.0% [95% CI, 40.7–63.3%] vs. 75.0% [95% CI, 65.3–84.7%]; *P* = 0.003).

Similarly, patients who did not achieve disease control had significantly larger CNAs than those who did; the median I-scores were 3405 (range, 377–28,520) 1024 (range, 256–23,380), respectively (*P* = 0.0017) (Fig. 4b). The I-score-high group (above the median; n = 75) had a significantly lower DCR than the I-score-low group (n = 76) (49.3% [95% CI, 38.0–60.6%] vs. 77.6% [95% CI, 68.2–87.0%]; *P* = 0.0003).

The cfDNA-high group had significantly worse TTP and OS than the cfDNA-low group; the median TTP values were 2.2 months (95% CI, 1.4–2.9) and 4.1 months (95% CI, 4.0–4.3), respectively (HR = 1.71 [95% CI, 1.20–2.44]; *P* = 0.002) (Fig. 4c), and the median OS values were 4.1 months (95% CI, 3.6–4.6) and 14.8 months (95% CI, 8.1–21.6), respectively (HR = 3.50 [95% CI, 2.36–5.20]; *P* < 0.0001) (Fig. 4d). Similarly, TTP and OS were significantly worse in the I-score-high group than in the I-score-low group;; the median TTP values were 2.2 months (95% CI, 1.5–2.8) and 4.1 months (95% CI, 3.9–4.3), respectively (HR = 2.09 [95% CI, 1.46–3.00]; *P* < 0.0001) (Fig. 4e), and the median OS values were 4.6 months (95% CI, 3.6–5.6) and 14.8 months (95% CI, 8.5–21.2), respectively (HR = 3.35 [95% CI, 2.24–5.01]; *P* < 0.0001) (Fig. 4f). When patients were divided into quartile groups according to the I-score, the median TTP values were 2.3 months (95% CI, 0.7–3.8; HR = 2.3), 2.0 months (95% CI, 1.0–2.9; HR = 2.1), 4.1 months (95% CI, 2.9–5.3; HR = 1.3), and 4.3 months (95% CI, 3.7–4.9; HR = 1.0) (*P* = 0.0025) (Fig. 4g), and the median OS value were 4.1 months (95% CI, 3.3–5.0; HR = 5.0), 5.2 months (95% CI, 2.9–7.5; HR = 3.2), 11.2 months (95% CI, 4.3–18.2; HR = 1.5), and 18.4 months (95% CI; 11.3–25.6; HR = 1.0) (*P* < 0.0001), for the highest, second-highest, second-lowest, and lowest quartile, respectively (Fig. 4h).

In the multivariable analysis of TTP after adjusting for the baseline AFP level, which was also associated with TTP in the univariable analysis, the I-score retained independent prognostic value (Table 2). In a multivariable analysis for OS that included the baseline AFP level, macroscopic vascular invasion, cfDNA concentrations, and I-score, which were significant in the univariable analysis, the cfDNA concentration, I-score, and AFP level remained statistically significant prognostic factors (Table 3). Patients with a higher cfDNA concentration showed a 2.51-fold (95% CI, 1.62–3.89; *P* < 0.0001) increased risk of death compared with those with a lower cfDNA concentration. Likewise, patients with a higher I-score showed a 1.85-fold (95% CI, 1.16–2.96; *P* = 0.010) increased the risk of death compared with those with a lower I-score.

**Table 2:** Univariable and multivariable analyses of TTP

			Univariable analysis		Multivariable analysis	
Variable		N	HR (95% CI)	*p* value	HR (95% CI)	*p* value
Age, years	< 65	125	1	0.736	–	–
	≥65	26	1.08 (0.69–1.70)		–	–
Sex	Male	137	1	0.806	–	–
	Female	14	0.93 (0.51–1.69)		–	–
ECOG PS	0	52	1	0.158	–	–
	1–2	99	1.31 (0.90–1.90)		–	–
Etiology	HBV	134	1	0.403	–	–
	Non-HBV	17	1.26 (0.73–2.17)		–	–
Child-Pugh class	A	140	1	0.595	–	–
	B	11	1.20 (0.68–2.38)		–	–
BCLC stage	B	5	1	0.191	–	–
	C	146	2.15 (0.68–6.76)		–	–
MVI	No	88	1	0.127	–	–
	Yes	63	1.32 (0.93–1.87)		–	–
EHS	No	15	1	0.090	–	–
	Yes	136	1.71 (0.92–3.20)		–	–
AFP (ng/mL)	< 200	73	1	0.008	1	0.092
	≥200	77	1.62 (1.14–2.31)		1.37 (0.95–1.98)	
cfDNA (ng/μL)	≤0.71^a)^	76	1	0.003	1	0.245
	> 0.71^a)^	75	1.71 (1.20–2.44)		1.27 (0.85–1.88)	
I-score	≤1637^a)^	76	1	< 0.0001	1	0.011
	>1637^a)^	75	2.09 (1.46–3.00)		1.71 (1.13–2.58)	

*HR* hazard ratio, *CI* confidence interval, *ECOG PS* Eastern Cooperative Oncology Group performance status, *HBV* hepatitis B virus, *BCLC* Barcelona Clinic Liver Cancer, *MVI* macroscopic vascular invasion, *EHS* extrahepatic spread, *AFP* alpha fetoprotein, *cfDNA* cell-free DNA

^a)^Median value

**Table 3:** Univariable and multivariable analyses of OS

			Univariable analysis		Multivariable analysis	
Variable		N	HR (95% CI)	*p* value	HR (95% CI)	*p* value
Age, years	< 65	125	1	0.734	–	–
	≥65	26	1.09 (0.67–1.77)		–	–
Sex	Male	137	1	0.678	–	–
	Female	14	0.86 (0.42–1.77)		–	–
ECOG PS	0	52	1	0.326	–	–
	1–2	99	1.23 (0.82–1.84)		–	–
Etiology	HBV	134	1	0.784	–	–
	Non-HBV	17	1.09 (0.60–1.98)		–	–
Child-Pugh class	A	140	1	0.118	–	–
	B	11	1.73 (0.87–3.43)		–	–
BCLC stage	B	5	1	0.383	–	–
	C	146	1.67 (0.53–5.26)		–	–
MVI	No	88	1	< 0.0001	1	0.125
	Yes	63	2.17 (1.48–3.18)		1.37 (0.92–2.06)	
EHS	No	15	1	0.775	–	–
	Yes	136	1.10 (0.57–2.11)		–	–
AFP (ng/mL)	< 200	73	1	< 0.0001	1	0.005
	≥200	77	2.27 (1.53–3.37)		1.80 (1.19–2.73)	
cfDNA (ng/μL)	≤0.71^a)^	76	1	< 0.0001	1	< 0.0001
	> 0.71^a)^	75	3.50 (2.36–5.20)		2.51 (1.62–3.89)	
I-score	≤1637^a)^	76	1	< 0.0001	1	0.010
	>1637^a)^	75	3.35 (2.24–5.01)		1.85 (1.16–2.96)	

*HR* hazard ratio, *CI* confidence interval, *ECOG PS* Eastern Cooperative Oncology Group performance status, *HBV* hepatitis B virus, *BCLC* Barcelona Clinic Liver Cancer, *MVI* macroscopic vascular invasion, *EHS* extrahepatic spread, *AFP* alpha fetoprotein, *cfDNA* cell-free DNA

^a)^Median value

Among the three, representative, specific patients in Fig. 3, the patient with the highest I-score (28,520) (Fig. 3b) had the worst treatment outcomes (median TTP, 1.2 months; median OS, 3.5 months), the patient with a middle I-score (7448) (Fig. 3c) had intermediate outcomes (median TTP, 4.2 months; median OS, 11.0 months), and the patient with the lowest I-score (500) (Fig. 3d) had the best outcomes (median TTP, 26.3+ months; median OS, 26.6+ months).

## Discussion

Based on genomic profiling using comprehensive high-throughput technologies, various molecular classifications were proposed in HCC [16–18]. Some of these molecular classifications have prognostic significance by classifying patients into favorable versus unfavorable prognosis groups after surgery; however, none has become a tangible tool in the clinical decision process because of the lack of validation and the scarcity of tissue in HCC. Furthermore, it remains unknown whether molecular subclasses and their prognostic value in surgically resected cases are preserved in unresectable HCCs subjected to systemic treatment. Therefore, there is a need to develop molecular prognostic biomarkers for advanced HCC patients receiving systemic therapy that are easily measured and address spatial and temporal tumor heterogeneity.

Tumor cfDNA is increasingly used as a biomarker in various cancers because of its potential to identify genomic alterations in tumor tissues and track the genomic evolution of metastatic tumors [19, 20]. In the present study, high pretreatment cfDNA levels in plasma were significantly associated with poor outcomes in advanced HCC patients receiving sorafenib. Patients with a higher cfDNA concentration were less likely to achieve disease control and more likely to die than those with a lower cfDNA concentration. These findings are consistent with those of previous studies in metastatic breast, ovarian, or non-small cell lung cancers, or melanoma, [19, 21–24], whereas they are inconsistent with those in metastatic colorectal or non-small cell lung cancers [25, 26]. These contradictory results could be attributed to different systemic treatments or cut-off values for cfDNA levels in the different studies.

CNA refers to a form of genomic structural variation and includes gene amplification, gain, loss, and deletion. CNAs affect a larger fraction of the genome in cancers than any other type of somatic genetic alteration and play a key role in cancer development and progression [27–29]. Previous studies reported both large-scale and focal chromosomal alterations in HCC, with a high level of copy number changes in oncogenes and tumor suppressors, or genes implicated in core cancer pathways including cell cycle, p53, phosphoinositide 3-kinase, mitogen-activated protein kinase, Wnt, and transforming growth factor beta signaling [30, 31]. Given that CNAs could result in genomic instability and increased genomic instability is associated with poor prognosis in multiple cancer types [32, 33], increased CNA rates across the genome are likely to be associated with poor prognosis. In this study, large genome-wide CNAs in pretreatment cfDNA was a significant independent indicator of poor TTP and OS in HCC patients receiving sorafenib. Patients with larger CNAs, as represented by a higher I-score, were more likely to have disease progression or death than those with smaller CNAs. Weiss et al reported that CNAs in plasma cfDNA indicated by copy number instability (CNI) scores were significantly higher in patients with diverse advanced cancers than noncancer controls, and the decrease in CNI scores from baseline could predict the response to systemic chemotherapy, immunotherapy, or combinations of both [34, 35]. Carter et al showed that baseline copy number profiling in circulating tumor cells could be used to classify chemo-sensitive versus chemo-refractory small cell lung cancer [36]. These results together with those of the present study suggest that CNAs in a liquid biopsy could serve as a prognostic or predictive indicator in advanced cancer patients receiving systemic therapy. However, since the present study was exploratory biomarker study with the exploratory nature of the analysis which also had a multiplicity issue, our study results should be validated in a well-designed prospective study with the appropriate statistical power for predefined endpoints.

To express genome-wide chromosomal instability, several scores such as CIN score [30], PA score [37], and S-score [38] were developed by the researchers. The CIN score was devised to measure the degree of CNAs across the entire genome of a tumor taking into account the total regions of the chromosome that are altered in a tumor as well as the amplitude of these alterations. The PA score was calculated as the number of SDs from the mean of the sum of the −log of the *P* values for the top five chromosome Z-scores of the 10 reference samples. S-score was calculated by the summation of all the squared Z-scores. The major difference between S-score and I-score is that I-score summates Z-scores which have more than 2 or less than − 2, not all the Z-scores. Many regions with Z-score less than 2 and more than − 2 can be detected in normal samples. However, by selection of highly deviated Z-scores in the I-score system, we could reflect definite cancer signals of ctDNA and reduce the noise which could occur during NGS experiments.

In addition to genome-wide CNA, we evaluated the association between *VEGFA* amplification in cfDNA and treatment outcomes based on a previous study suggesting *VEGFA* genomic amplification in HCC tumor tissues as a predictive biomarker for sorafenib [6, 8]. Although *VEGFA* copy number was significantly higher in HCC patients than in healthy controls, a significant association between *VEGFA* copy number and sorafenib treatment outcomes was not observed. However, since *VEGFA* amplification was evaluated only in part of the study population because of the limited quantity of blood sample in each patient, which could be a potential bias, further investigation is required to validate the predictive value of *VEGFA* amplification in HCC treated with sorafenib.

## Conclusions

In conclusion, we demonstrated that pretreatment concentration and genome-wide CNAs in cfDNA are potential biomarkers predicting treatment outcomes in advanced HCC patients receiving first-line sorafenib.

## Additional files

Additional file 1: **Figure S1.** Scatter plot demonstrating the correlation of the I-score with the total cell-free DNA concentration. **Figure S2.** Kaplan-Meier curves for (**A**) time to progression and (**B**) overall survival according to the VEGFA ratio. Abbraviation: VEGFA, vascular endothelial growth factor-A. (DOCX172 Kb)

## Abbreviations

AFP: Alpha-fetoprotein
cfDNA: Cell-free DNA
CI: Confidence interval
CNA: Genome-wide copy number alteration
CNI: Copy number instability
CR: Complete response
ctDNA: Circulating tumor DNA
DCR: Disease control rate
ddPCR: Digital polymerase chain reaction
HCC: Hepatocellular carcinoma
HR: Hazard ratio
OS: Overall survival
PD: Progressive disease
RECIST: Response Evaluation Criteria In Solid Tumors
TTP: Time to progression
VEGF: Vascular endothelial growth factor
