# Supplementary material for: Genome-wide copy number alteration and VEGFA amplification of circulating cell-free DNA as a biomarker in advanced hepatocellular carcinoma patients treated with Sorafenib
Source: BMC Cancer. 2019 Apr 1;19:292. doi: 10.1186/s12885-019-5483-x (PMC6444867; doi:10.1186/s12885-019-5483-x)
Supplement: Supplementary file 1 — Figure S1. Scatter plot demonstrating the correlation of the I-score with the total cell-free DNA concentration. Figure S2. Kaplan-Meier curves for (A) time to progression and (B) overall survival according to the VEGFA ratio. Abbraviation: VEGFA, vascular endothelial growth factor-A. (DOCX172 Kb) [file 12885_2019_5483_MOESM1_ESM.docx]

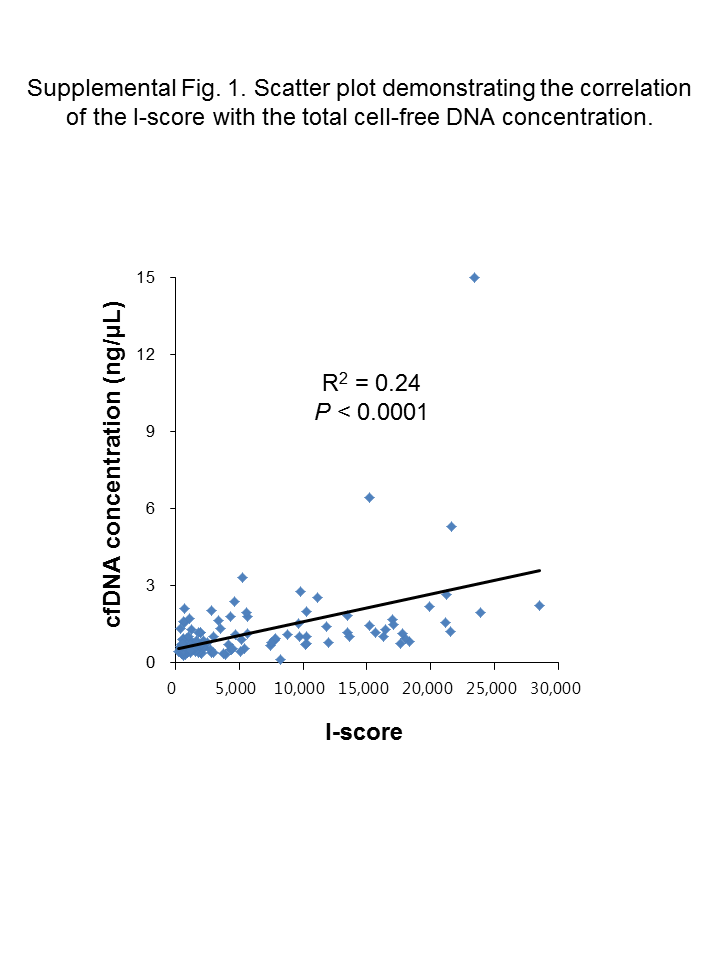
Supplemental Fig. 1. Scatter plot demonstrating the correlation of the I-score with the total cell-free DNA concentration.


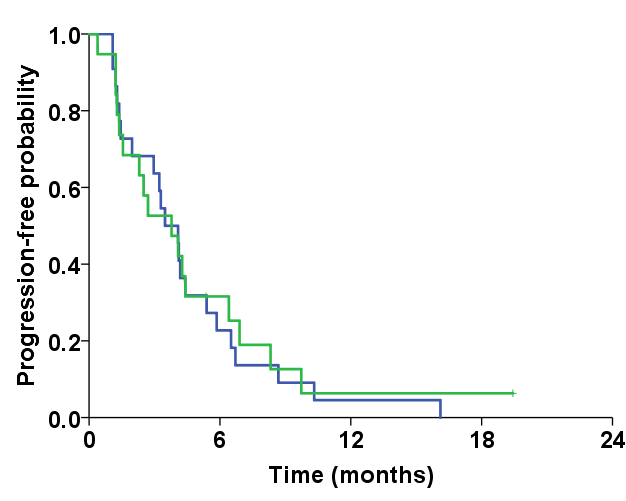


**A**

**B**

***p* = 0.781**


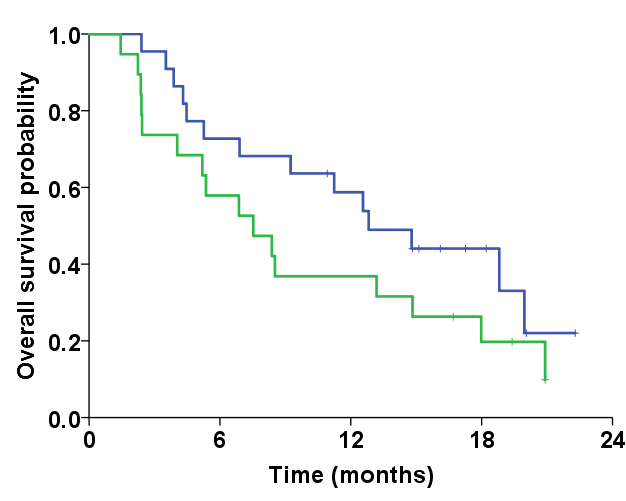


***p* = 0.180**

VEGFA ratio-low (≤median)

VEGFA ratio-high (>median)

VEGFA ratio-low (≤median)

VEGFA ratio-high (>median)

Supplemental Fig. 2. Kaplan-Meier curves for (A) time to progression and (B) overall survival according to the VEGFA ratio. VEGFA, vascular endothelial growth factor-A.
